# Burden of Informal Caregivers from an HHC Military Hospital in Riyadh, Saudi Arabia: A Cross-Sectional Study

**DOI:** 10.3390/ijerph22020313

**Published:** 2025-02-19

**Authors:** Daniela Patrícia Santos Costa, Husam I. Ardah, Amjad Searya

**Affiliations:** 1 Home Health Care, Ministry of National Guard—Health Affairs, Riyadh 11426, Saudi Arabia; 2 King Abdullah International Research Center, Riyadh 11426, Saudi Arabia; ardahhu@mngha.med.sa; 3 King Saud Bin Abdulaziz University for Health Sciences, Riyadh 11426, Saudi Arabia; searyaa@mngha.med.sa

**Keywords:** informal caregiver burden, Zarit scale, Saudi Arabia, home health care, palliative, cancer, dementia, chronic

## Abstract

(1) Background: Home health care (HHC) services guarantee good patient care and family support. Understanding how we can better help our informal caregivers (ICs) by understanding their needs and the burden they experience is crucial. (2) Methods: A cross-sectional study was conducted from June to September 2024 at an HHC facility in Riyadh, Saudi Arabia. The 22-item Zarit Burden Interview (ZBI-22) was used to assess the caregiver burden (CB). ICs of patients with specific diseases (palliative, cancer, chronic, and dementia patients) and other factors were studied to identify any association with informal caregiver burden (ICB). (3) Results: Significant CB was defined as ZBI ≥ 21. The IC (384) participants comprised 119 caregivers of dementia patients, 104 caregivers of chronic patients, 83 caregivers of palliative patients, and 78 caregivers of cancer patients. The total mean of the ZBI-22 score among ICs was 31.66, representing a significant CB. The means by groups were as follows: chronic patients, 38.28; dementia patients, 34.97; cancer patients, 25.21; and palliative patients, 22.94. Other variables, such as the duration of care, education level, relationship with the patient, and unemployment, were associated with CB (*p*-value < 0.05). (4) Conclusions: The caregivers’ characteristics and the patients’ disease were associated with significant CB. More interventions from healthcare workers to support ICs are essential to release their burden.

## 1. Introduction

Population aging is a phenomenon all over the world; even though the Kingdom of Saudi Arabia has a relatively young average population compared with other developed countries, it is following the same trend. This is supported by the United Nations state report that recently showed that the percentage of elderly individuals in the KSA will witness a sharp increase in the next three decades (5.6% in 2017 to 23% by 2050) [1,2]. This scenario suggests a high potential for an increased prevalence of comorbidities, including chronic diseases, cancer, and dementia. As a result, patients requiring palliative care will need close and continuous monitoring [3].

According to these facts, it is essential to be aware that the increment in patients institutionalized will be more frequent. Some research suggests that most people over sixty-five years old want to live in their houses for as long as possible. Additional support is necessary through HHC services to avoid institutionalization and keep individuals in their homes [4].

HHC consists in a system of qualified practitioners who provide care to patients in their homes, such as nurses, physicians, occupational therapists, dietitians, and social services [5]. The purpose of HHC services is to promote an optimal level of well-being for the patients, to help and support them to improve and maximize independence in their daily activities, and to assist in their needs to avoid hospitalization or admission to long-term facilities [6,7]. In Saudi Arabia, the first HHC program was established in 1991 by the King Faisal Specialized Hospital and was only for terminal cancer patients. Later, in 1995, the Ministry of National Guard Health Affairs (MNGHA) established the HHC program to reduce the length of hospital stays [8]. Our HHC services are provided according to patients’ interventions, regardless of whether there is family support or not. The HHC work schedule is from 7 a.m. to 11 p.m., seven days a week. The only exception is palliative and cancer patients, where specialized services are provided only five days a week by an interdisciplinary team of physicians and nurses.

As the elderly population grows, and considering the strong Middle Eastern culture of “taking care of your own”, informal caregivers play a crucial role in providing diverse care. Their involvement is essential in supporting the elderly within their home environments [9]. Informal caregivers, who can often be described as family or friends, provide unpaid support to individuals with chronic conditions or disabilities, helping with daily activities such as bathing, dressing, eating, administering medication, and mobility, while also offering emotional and social support [10]. Their role is crucial in the healthcare system, particularly with the aging population and the increase in chronic diseases. Even though providing informal care can be described as fulfilling and rewarding, it can lead to physical, emotional, and financial stress [11,12,13]. The HHC program complements this role by providing visits from healthcare professionals who deliver specialized medical care, such as wound care, catheter insertion, and endovenous medication. Informal and formal caregivers work together to provide comprehensive care. Informal caregivers offer continuous, day-to-day support at home, while formal caregivers provide clinical expertise and supervision to alleviate the burden on informal caregivers. The term “burden” in the context of caregiving involves a complex interplay. It could refer to the physical, emotional, financial, and psychological strain experienced by caregivers as a result of their caregiving responsibilities. Recognizing and addressing these burdens is essential for improving both caregiver and patient outcomes [14,15].

This paper proposes to assess the burden experienced by informal caregivers of patients receiving HHC services at the NGHA through a cross-sectional study. The aim is to compare the caregiver burden across four different patient comorbidities, including palliative, cancer, chronic, and dementia patients. In general, the findings of this study are intended to enhance our understanding of the challenges informal caregivers face, according to the burden level, and contribute to developing a more supportive environment for caregivers and care recipients. Through the HHC program, we can offer increased support by scheduling additional visits from our healthcare professionals; that way, it alleviates the caregiver burden and promotes greater teamwork. In summary, measuring the burden of informal care helps address caregivers’ needs and improve patient care and the overall quality of care.

A systematic literature review was conducted prior to selecting the tool for interviewing informal caregivers. Factors such as the patient’s age, the population of care recipients, the content of the questions, and the tool’s validity and internal consistency were considered. Based on these considerations, a suitable tool that met all the study’s requirements was identified.

The Zarit Burden Interview 22-item (ZBI-22) short Arabic version was the selected tool used to assess the informal caregiver burden. The survey was part of a comprehensive understanding of the impact of the constantly rising burden on informal caregivers. Thus, translating valid and reliable tools for measuring CB is crucial. The results are expected to provide a factual evidence base for measuring and improving the quality of the HHC program of the NGHA of Saudi Arabia and similar HHC programs all over the country.

## 2. Materials and Methods

### 2.1. Study Design and Setting

This study is a cross-sectional survey on informal care. It uses the Arabic version of the Zarit Burden Interview (ZBI), a short version of 22 questions, to assess the burden of informal caregivers. This study was conducted within the HHC program of the National Guard Health Affairs in King Abdulaziz Medical City in Riyadh, one of the largest hospitals in the central region of Saudi Arabia, which receives cases from all social and economic classes. This HHC program provides regulated care through various healthcare professionals in patients’ homes. The mission is to offer comprehensive healthcare support, including nursing, medical, and social support, to patients and their families in a home environment. The goal is to meet patients’ needs (physical, psychological, and social), ensuring a good quality of life and promoting comfort, safety, and social value for all.

### 2.2. Participants and Data Collection

The entire population enrolled in the HHC program who were available during the home visits and willing to participate in the study survey were considered. The Most Responsible Physician (MRP) refers patients to the HHC program. Once patients meet the program criteria, they are admitted, and their information is automatically entered into our electronic database. From the first nurse visit to the patient’s home, the duration of their participation in the HHC program is tracked in our information system, “Best Care”. This allows us to monitor the length of their involvement in the program.

The inclusion criteria comprised all informal caregivers aged equal to or above 18, male and female, Arabic speakers, only one caregiver per patient, and a literate person taking care of the patient continuously for a minimum of 4 weeks. This selection ensured that only respondents who had been leading informal care for more than 4 weeks for patients with and highly dependent on care were gathered, as well as all patients with one of these comorbidities (palliative, cancer, chronic, or dementia). All caregivers wanted to participate in the survey. From a total population of 1080 patients, 415 met the criteria, but only 384 completed the survey. Caregivers who did not meet the eligibility criteria were excluded. Three hundred and eighty-four respondents completed the questionnaire on informal care.

Data were collected during June and September 2024 using the Arabic version of the short 22-item ZBI-22 questionnaire for measuring ICB, obtained from Mapi Research Trust, Lyon, France (https://eprovide.mapi-trust.org/, accessed on 7 May 2024). Originally developed to assess the burden on caregivers of dementia patients, the Zarit Burden Interview (ZBI) has become one of the most widely used tools for measuring caregiver burden (CB). It has shown strong construct validity and high internal reliability and has been extensively validated and studied. The ZBI is user-friendly and demonstrates consistent internal reliability, with Cronbach’s alpha values consistently above 0.8 in numerous studies [16,17].

The process of collecting caregivers’ responses involved some key steps. Initially, caregivers who met the pre-established criteria were selected for the study. During the nurses’ HHC visits, the caregivers were meticulously informed about the purpose of the survey and its role in the research. Then, if caregivers agreed to participate, they were asked to sign a consent form. Once the consent was obtained, a link to the survey was sent to the caregiver via WhatsApp, which was done to minimize any potential bias. Additionally, the confidentiality of the study was carefully explained to the caregivers to ensure that their responses would remain anonymous and secure. Automatically, the answers were sent to Google Drive. Before concluding the survey, all answers had to be completed, and participants could not leave any answer without a response before sending the link.

### 2.3. Measurements

The survey was composed of 2 sections. The first section included the sociodemographic details of the participants, such as age, gender, education level, marital status, employee status, relationship with the care recipient, and the time since caregiving. Section two of the survey was the ZBI-22 questionnaire that included the impact of caregiving on distinct aspects (caregiver’s physical health, psychological well-being, finances, and social life, and the relationship between the caregiver and patient). Each question is scored on a five-point Likert scale: never (0), rarely (1), sometimes (2), quite frequently (3), and nearly always (4). Total scores range from 0 to 88. The cut-off values are as follows: 0–20, little or no burden; 21–40, mild to moderate burden; 41–60, moderate to severe burden; and 61–88, severe burden [18]. The scale permits a prompt evaluation and provides a significant understanding of the informal caregiver’s burden, facilitating profitable data collection and analysis.

The survey was identical for all informal caregivers. The only way to differentiate each group was by the order of the first question.

Before sending the survey, the group each participant belonged to according to the patient’s disease (palliative, cancer, chronic, or dementia) was selected. The survey took around 5 min to answer.

### 2.4. Statistical Analysis

All categorical data obtained from the survey were calculated and presented as numbers and percentages. Continuous data were presented as means, standard deviations, medians, and quartiles. As appropriate, inferential statistics, such as the *t*-test and chi-square test, were used to compare variables. Multivariable logistic regression analysis was used to identify any association between burden scores, patient condition, and informal caregiver characteristics.

All data were entered into and analyzed through SAS 9.4 (SAS Institute Inc., Cary, NC, USA).

### 2.5. Ethical Considerations

The Department Research Committee of the NGHA reviewed and approved the ethical approval. The respondents were informed that participation was voluntary and that all information and data would remain confidential and anonymous. The respondents needed to provide written informed consent at the start and end of the survey.

## 3. Results

### 3.1. Questionnaire

#### 3.1.1. Sociodemographic Characteristics of Study Participants

The information regarding our sample (*n* = 384), i.e., informal caregivers’ sociodemographic characteristics, is shown in Table 1. The mean (SD) age of the informal caregivers was 52.3 (11.49) years, ranging from 19 to 85 years. Regarding the type of care recipients received according to their morbidity, 21.6% were palliative, 20.3% had cancer, 27.1% had a chronic disease, and 31% had dementia. On average, informal caregivers provided care for more than 3 years (41.7%). Half (50%) of the respondents had a general level of education, and less than one-third (27.3%) had jobs. More than half (69.3%) of the participants were female, and the majority (75%) were married. Regarding the relationship with the patients, more than half of them lent care to their parents (58.1%).

#### 3.1.2. The ZBI-22 Tool

The prevalence of burden among caregivers was reported using the ZBI-22 questionnaire tool by mean and frequency. The total ZBI-22 score ranged from 0 to 88. The mean ZBI-22 score was 31.66. Using the ZBI-22 cutoff score of 21, 74.48% had significant CB (286 ≥ 21). Regarding the individual items of the ZBI, the highest score was for the question, “Do you feel your relative is dependent upon you?”, with a mean (SD) of 2.5 (1.13). The lowest score was for the question, “Do you feel that your relative currently affects your relationship with other family members or friends in a negative way?”, with a mean (SD) of 0.9 (1.10). Details of the ZBI-22 item characteristics are presented in Table 2, for all 22 questions of the short version, with the respective means and standard deviations for the sample (*n* = 384).

### 3.2. Informal Caregiver Burden

**Figure 1:**
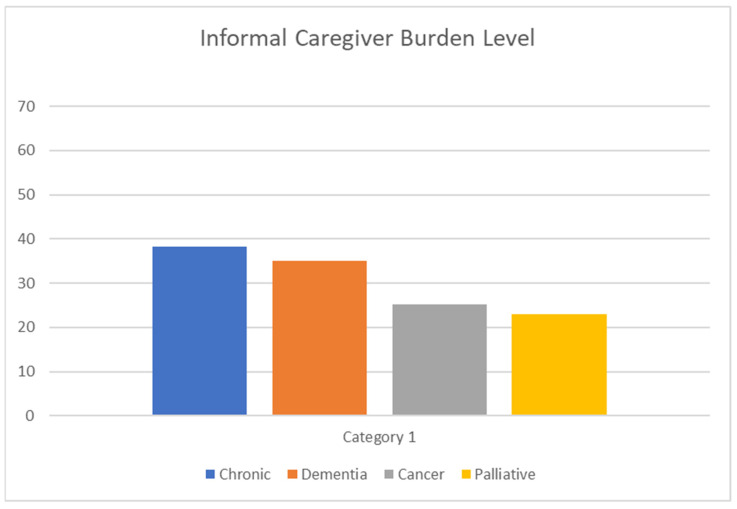
Severity of burden among informal caregivers by recipients’ conditions.

From Figure 1, it is possible to see the frequencies and compare the differences in ICB according to the recipients’ diseases.

### 3.3. Bivariate Analysis (ZBI-22) with Significant p-Value > 0.05

The relationship between CB and the variables studied in the bivariate analysis is described in Table 3. In total, there were 286 responses corresponding to Yes (there is a burden) (ZBI-22 scored ≥ 21), and 98 responses corresponding to No (there is no burden) (ZBI-22 scored < 21). The caregiver’s age was significant to the CB (*p*-value 0.0003). The oldest persons had the highest burden compared with the youngest ones. The bivariate analysis for factors associated with CB revealed that the caregivers taking care of patients with chronic disease (32.9%) and dementia (35.3%) had a high burden (ZBI-22 score ≥ 21) compared with those taking care of patients under palliative care (15.7%) and with cancer (16.1%). The duration of care reveals that the caregivers with a short duration of caregiving of < 1 year (26.2%) had a lower burden compared with those with > 3 years (44.4%). Regarding the education level (*p*-value 0.0113), those with general education (51.7%) had a greater burden compared with caregivers with university education (39.2%). Also, whether the caregivers had a job impacted their burden, and it is possible that those working had a lower burden (20.3%) compared with those who were unemployed (79.7%). The gender variable also showed that females had a higher burden (76.2%). Relationship with the patient as a husband/wife demonstrated significance (*p*-value < 0.0001) to ICB. Marital status was not significantly associated with CB (*p*-value > 0.05).

### 3.4. Multiple Logistic Regression Analysis

Multiple logistic regression analysis (Table 4) showed that only four factors were independently associated with ICB. The CB was statistically significant for caregivers of patients with dementia compared with those of patients with cancer, and the same for caregivers without a job. Caregivers with high education levels had a significant burden compared with those with a university education. Also, the burden was substantial for those caring for spouses compared with those caring for their parents.

## 4. Discussion

This study, conducted from a military hospital in Riyadh, Saudi Arabia, aims to determine the prevalence of burden among different patient caregivers and identify the associated risk factors. The ZBI-22 was used to assess the degree of burden. We observed that respondents who took care of chronic and dementia patients had the highest level of burden, compared with carers who took care of cancer and palliative patients, who had the lowest level of burden. The mean ZBI-22 score in this study was 25.21 for cancer patients, similar to that reported in a study from the United Kingdom that included cancer patients, and the mean ZBI-22 score was 23 [19]. Equally, the mean ZBI-22 score was 22.94 for palliative patients, the same as in a study on palliative care in Malaysia, where the ZBI-22 mean score was 23 [20]. The ZBI scale has been used extensively in caregivers of patients with chronic illness [21,22]. Informal care is often essential to the care provided to patients, especially those with chronic diseases. Recent studies have shown that many informal caregivers experience a substantial burden from their caregiving tasks. It has been demonstrated that caregiving amongst the elderly is an independent risk factor for morbidity and mortality [23], which supports the high level of the mean burden for chronic patients, with a score of 38.29. Likewise, Gratao et al. observed a significant relationship between a caregiver’s burden and the recipient’s stage of dementia [24], which corroborates with the burden level from our study, in which the mean score was 34.97. Denno et al. concluded that as the burden of caregivers increased, the more likely they were to experience anxiety and depression [25]. Against possible expectations, palliative and cancer patients’ caregivers reported lower levels of burden when compared with others. These facts could be related to the excellent support and good services provided in our HHC department (5 days a week) by the palliative doctors, together with the nurses going for patient visits, providing specialized care, and consequently, all these components could be associated with an excellent factor that helps informal caregivers in their tasks with close monitoring and supporting the patient’s needs. Also, to our knowledge, the present study is the first in Saudi Arabia to explore the burden of informal caregivers of different patient diseases and compare the burden between them. Evidence in the literature indicates that cultural factors influence caregivers’ burden, with one study reporting that these aspects explained 29% of the variance in burden [26]. Al-Khashan et al. asserted that home services that provide additional healthcare support to patients improve the self-confidence of caregivers, and these frequent home visits also increase caregiver satisfaction [27]. The results will provide a basis for developing a program to teach and support this group of caregivers in their arduous work.

We detailed the results of the responses of the ICs to the 22 items of the ZBI-22 to quickly identify which questions had more impact on the ICB and maybe serve as a guide for future research/interventions to minimize the ICB in our HHC department. Only 4 items out of 22 scored, on average, >2 on the 0–4 scale. This may be a target for interventions dealing with ICB. Another example is the question, “Do you feel your relative is dependent upon you?”, which scored an average of 2.5 on the 0–4 scale, which represents the idea that the patient depends on them, describing the feeling of the total responsibility of the total care, which causes even more anxiety for the IC. Meeting the information requirements of caregivers supporting them psychosocially may reduce their fears, stress, and, subsequently, the burden they suffer.

The caregivers’ mean age in the present study was 52.3 ± 11.49 years, following those reported in previous studies [28,29,30], as the caregivers’ ages ranged between 43.8 and 63.1 years. In our study, older caregivers experienced a higher burden than younger ones, which could be explained by the culture in Saudi Arabia, where the responsibilities increase naturally according to a person’s age, which causes more physiological stress. The current study also showed that the burden was higher among participants with lower education, who were unemployed and had the most extended duration of care. Regarding the duration, time is reasonably expected for this association. In South Korea, Yoon et al. [31] reported that a longer time spent providing care per day was significantly associated with caregiver burden.

The relationship between the caregiver and recipient was also a decisive factor, and as reported in previous studies, husbands and wives noticed a significant burden; the burden was greater among spouses [32,33].

Females had a higher burden compared with males; studies suggest that women caregivers are usually involved in physically demanding tasks (e.g., bathing, feeding, and dressing) compared with their male counterparts, who are more likely to provide financial support [34].

Previous studies identified factors that determine the presence and degree of ICB, such as the duration of care, the education level, and whether the caregiver is employed [35,36,37,38]. This study shows only four variables independently associated with significant CB: patient disease, and caregivers who are unemployed, have high education, and take care of spouses; the other factors have no significant association with CB.

Many different factors can influence CB, such as culture and beliefs, like religion. Other concepts and beliefs can affect caregiving expectations and behaviors, motivations to provide care, and different concepts of caregiver distress or burden [39]. Differences in some studies could be attributed to the various carers’ sociodemographic characteristics, tools utilized, methodology, and cultural norms [40].

While this study offers valuable insights, several significant limitations should be addressed in future research. First, this study was conducted in a single healthcare facility, limiting the findings’ results. Additionally, the small sample size for specific patient groups, particularly palliative and cancer patients, might have reduced this study’s statistical power. Larger sample sizes are necessary for a more comprehensive understanding of caregiver burden across various patient conditions. Furthermore, this study did not assess crucial factors influencing caregiver burden, such as the caregiver’s mental health status, social support, or access to resources. Given the unique cultural context of Saudi Arabia, future research should examine how cultural values and social norms influence the caregiving experience, particularly regarding expectations and perceived responsibilities [41,42,43].

Notwithstanding these limitations, this study strongly impacts the focus on exploring the magnitude of ICB and identifying the factors associated with a higher burden, leading to a better-targeted intervention by healthcare practitioners and researchers.

Specific recommendations for reducing caregiver burden and enhancing support should be addressed. The following healthcare interventions should be prioritized: Psychosocial Support: establishing programs that offer counseling and stress management can help reduce anxiety and depression among caregivers. Educational Resources: providing training programs focused on disease management and caregiving techniques can enhance caregivers’ confidence and competence. Integrated Healthcare Support: ensuring caregivers feel comfortable contacting healthcare professionals from the HHC program for practical advice and emotional support related to caregiving tasks is essential. Family Support: strengthening social and family support networks is crucial, as well as providing informal caregivers with the resources necessary to manage their caregiving responsibilities more effectively.

## 5. Conclusions

The ZBI, which provides a comprehensive assessment of both objective and subjective burden, was designed for caregivers of patients with dementia. Even so, it is one of the most commonly used burden measures. It has been validated in many culturally or ethnically different populations and is widely used with a wide variety of other disorders, including cancer, palliative care, and chronic illness.

In conclusion, the burden of ICs had a more significant impact on patients with chronic disease and dementia than on those under cancer and palliative care. This could be attributed to the specialized services for palliative and cancer patients. Therefore, healthcare staff must be more aware of patients’ and their families’ experiences and potential needs. Eventually, an interdisciplinary approach and collaborative visits with physicians and nurses could minimize ICB.

For future research, we suggest conducting longitudinal studies on caregiver burden. Such studies would provide a deeper understanding of how caregiver burden develops over time and its impact on caregivers’ well-being, particularly for those caring for patients with progressive conditions like dementia and chronic diseases. Furthermore, exploring the impact of specific caregiving tasks could be valuable. Tasks such as administering medications, managing behavioral symptoms of dementia, and performing physical care activities (e.g., bathing and dressing) may cause variable levels of stress depending on the nature of the illness. Investigating these nuances could lead to more targeted and effective caregiver support strategies. Finally, assessing the effectiveness of interventions through randomized controlled trials could help identify the most effective approaches for alleviating caregiver burden and improving their quality of life.

## Figures and Tables

**Table 1:** Sociodemographic characteristics of study participants.

Variable	Category	Total
Age	*n*	384
Age	Mean (SD)	52.3 ± 11.49
Age	Median (Q1, Q3)	53.0 (45.50, 60.00)
Age	Min, Max	19.0, 85.0
Disease	Chronic	104 (27.1%)
Disease	Dementia	119 (31.0%)
Disease	Cancer	78 (20.3%)
Disease	Palliative	83 (21.6%)
Duration of Care	Less one year	114 (29.7%)
Duration of Care	More than three years	160 (41.7%)
Duration of Care	One year to three years	110 (28.6%)
Education Level	General education	192 (50.0%)
Education Level	High education	28 (7.3%)
Education Level	University education	164 (42.7%)
Employee	No	279 (72.7%)
Employee	Yes	105 (27.3%)
Gender	Female	266 (69.3%)
Gender	Male	118 (30.7%)
Marital Status	Divorced	30 (7.8%)
Marital Status	Married	288 (75.0%)
Marital Status	Single	60 (15.6%)
Marital Status	Widowed	6 (1.6%)
Relationship with Patient	Brother/Sister	50 (13.0%)
Relationship with Patient	Father/Mother	223 (58.1%)
Relationship with Patient	Grandmother/Grandfather	19 (4.9%)
Relationship with Patient	Husband/Wife	65 (16.9%)
Relationship with Patient	Other	27 (7.0%)

Total responses of caregivers (384).

**Table 2:** Responses of informal caregivers to the ZBI-22 items.

Item (384 Responses)	0 (Never)	1 (Rarely)	2 (Sometimes)	3 (Quite Frequently)	4 (Nearly Always)	Mean (SD)
1. Are you afraid of what the future holds for your neighbor?	95 (24.7%)	75 (19.5%)	99 (25.8%)	98 (25.5%)	17 (4.4%)	1.7 ± 1.23
2. Are you ashamed of your relative’s behavior?	201 (52.3%)	62 (16.1%)	65 (16.9%)	56 (14.6%)	0 (0%)	0.9 ± 1.13
3. Do you feel angry or angry if you are with your relative?	202 (52.6%)	62 (16.1%)	83 (21.6%)	34 (8.9%)	3 (0.8%)	0.9 ± 1.08
4. Do you feel that more needs to be done than you already do for your neighbor?	45 (11.7%)	67 (17.4%)	121 (31.5%)	102 (26.6%)	49 (12.8%)	2.1 ± 1.19
5. Do you feel that the time you spend with your relative affects your time?	95 (24.7%)	91 (23.7%)	112 (29.2%)	68 (17.7%)	18 (4.7%)	1.5 ± 1.18
6. Do you feel that you are missing privacy to some degree because of your relative?	177 (46.1%)	72 (18.8%)	79 (20.6%)	50 (13.0%)	6 (1.6%)	1.1 ± 1.15
7. Do you feel that you can improve the level of care you provide to your relative?	29 (7.6%)	82 (21.4%)	117 (30.5%)	109 (28.4%)	47 (12.2%)	2.2 ± 1.12
8. Do you feel that you don’t have enough money to take care of your relative in addition to the rest of your allowance?	74 (19.3%)	54 (14.1%)	124 (32.3%)	106 (27.6%)	26 (6.8%)	1.9 ± 1.20
9. Do you feel that you have lost control of your life since your relative’s illness?	129 (33.6%)	53 (13.8%)	98 (25.5%)	86 (22.4%)	18 (4.7%)	1.5 ± 1.29
10. Do you feel that your health has been affected by the result of your neighbor’s care?	130 (33.9%)	75 (19.5%)	114 (29.7%)	56 (14.6%)	9 (2.3%)	1.3 ± 1.15
11. Do you feel that your relative is currently affecting your relationship with the rest of the family or friends negatively?	199 (51.8%)	70 (18.2%)	68 (17.7%)	45 (11.7%)	2 (0.5%)	0.9 ± 1.10
12. Do you feel that your relative expects to take care of him as if you are the only person who can rely on him?	46 (12.0%)	52 (13.5%)	114 (29.7%)	126 (32.8%)	46 (12.0%)	2.2 ± 1.18
13. Do you feel that your relative is asking for help and help more than he really needs?	130 (33.9%)	100 (26.0%)	61 (15.9%)	48 (12.5%)	45 (11.7%)	1.4 ± 1.37
14. Do you feel that your social life is suffering because of your care for your relative?	137 (35.7%)	87 (22.7%)	81 (21.1%)	67 (17.4%)	12 (3.1%)	1.3 ± 1.21
15. Do you feel the psychological pressure resulting from the distribution of attention between the care of your relative and the performance of your responsibilities towards the family or work?	78 (20.3%)	107 (27.9%)	110 (28.6%)	63 (16.4%)	26 (6.8%)	1.6 ± 1.18
16. Do you feel tight (uncomfortable) when you’re with your relative?	170 (44.3%)	63 (16.4%)	95 (24.7%)	48 (12.5%)	8 (2.1%)	1.1 ± 1.17
17. Do you feel uncomfortable inviting a friend because of your relative?	163 (42.4%)	70 (18.2%)	91 (23.7%)	48 (12.5%)	12 (3.1%)	1.2 ± 1.19
18. Do you feel unsure of what to do with your relative?	131 (34.1%)	70 (18.2%)	108 (28.1%)	70 (18.2%)	5 (1.3%)	1.3 ± 1.16
19. Do you feel you can’t continue to take care of your relative?	180 (46.9%)	71 (18.5%)	77 (20.1%)	47 (12.2%)	9 (2.3%)	1.0 ± 1.17
20. Do you price that your relative depends on you?	27 (7.0%)	49 (12.8%)	92 (24.0%)	148 (38.5%)	68 (17.7%)	2.5 ± 1.13
21. In general, how much do you feel the burden of caring for your neighbor?	117 (30.5%)	76 (19.8%)	117 (30.5%)	64 (16.7%)	10 (2.6%)	1.4 ± 1.16
22. Would you like to leave your neighbor’s care for someone else?	193 (50.3%)	59 (15.4%)	76 (19.8%)	50 (13.0%)	6 (1.6%)	1.0 ± 1.17

Zarit Burden Interview 22-item (ZBI-22) short version in English.

**Table 3:** The relationship between caregiver burden and the studied variables.

Name of Former Variable	Cat2	Yes (ZBI-22 Score ≥ 21) = 286	No (ZBI-22 Score < 21) = 98	Total = 384	*p*-Value
Age	Mean (SD)	53.5 (10.97)	48.8 (12.29)	52.3 (11.49)	0.0003 ^
Age	Median (Q1, Q3)	54.0 (48.00, 61.00)	50.0 (40.00, 57.00)	53.0 (45.50, 60.00)	0.0003 ^
Disease	Chronic	94 (32.9)	10 (10.2)	104 (27.1)	<0.0001 **
Disease	Dementia	101 (35.3)	18 (18.4)	119 (31.0)	<0.0001 **
Disease	Cancer	46 (16.1)	32 (32.7)	78 (20.3)	<0.0001 **
Disease	Palliative	45 (15.7)	38 (38.7)	83 (21.6)	<0.0001 **
Duration of Care	Less than one year	75 (26.2)	39 (39.8)	114 (29.7)	0.0342 **
Duration of Care	More than three years	127 (44.4)	33 (33.7)	160 (41.7)	0.0342 **
Duration of Care	One year to three years	84 (29.4)	26 (26.5)	110 (28.6)	0.0342 **
Education Level	General education	148 (51.7)	44 (44.9)	192 (50.0)	0.0113 **
Education Level	High education	26 (9.1)	2 (2.0)	28 (7.3)	0.0113 **
Education Level	University education	112 (39.2)	52 (53.1)	164 (42.7)	0.0113 **
Employee	No	228 (79.7)	51 (52.0)	279 (72.7)	<0.0001 **
Employee	Yes	58 (20.3)	47 (48.0)	105 (27.3)	<0.0001 **
Gender	Female	218 (76.2)	48 (49.0)	266 (69.3)	<0.0001 **
Gender	Male	68 (23.8)	50 (51.0)	118 (30.7)	<0.0001 **
Marital Status	Divorced	21 (7.3)	9 (9.2)	30 (7.8)	0.4497 ^^
Marital Status	Married	220 (76.9)	68 (69.4)	288 (75.0)	0.4497 ^^
Marital Status	Single	41 (14.4)	19 (19.4)	60 (15.6)	0.4497 ^^
Marital Status	Widowed	4 (1.4)	2 (2.0)	6 (1.6)	0.4497 ^^
Relationship with Patient	Brother/Sister	42 (14.7)	8 (8.2)	50 (13.0)	<0.0001 **
Relationship with Patient	Father/Mother	149 (52.1)	74 (75.5)	223 (58.1)	<0.0001 **
Relationship with Patient	Grandmother/Grandfather	10 (3.5)	9 (9.2)	19 (4.9)	<0.0001 **
Relationship with Patient	Husband/Wife	62 (21.7)	3 (3.1)	65 (16.9)	<0.0001 **
Relationship with Patient	Other	23 (8.0)	4 (4.0)	27 (7.0)	<0.0001 **

Denominator of the percentage is the total number of subjects in each group. *t*-Test/^ Wilcoxon rank sum test is used to calculate the *p*-value. ** Chi-square test is used to calculate the *p*-value. ^^ Fisher exact test is used to calculate the *p*-value.

**Table 4:** Multivariable logistic regression modeling the relationship between caregiver burden and the studied variables.

Effect	Beta	Standard Error	Odds Ratio	[95% Conf. Interval]	*p*-Value
Age	−0.00114	0.0144	0.999	(0.97, 1.03)	0.9367
**Disease** Chronic **vs.** Dementia	0.5697	0.4459	1.768	(0.74, 4.24)	0.2014
**Disease** Cancer **vs.** Dementia	−0.8910	0.4259	0.410	(0.18, 0.95)	0.0364
**Disease** Palliative **vs.** Dementia	−0.8242	0.4238	0.439	(0.19, 1.01)	0.0518
**Gender** Female **vs.** Male	0.4141	0.3033	1.513	(0.83, 2.74)	0.1721
**Duration of Care** More than three years **vs.** Less than one year	0.2763	0.3544	1.318	(0.66, 2.64)	0.4357
**Duration of Care** One year to three years **vs.** Less than one year	0.2582	0.3596	1.295	(0.64, 2.62)	0.4727
**Marital Status** Divorced **vs.** Married	0.2789	0.4827	1.322	(0.51, 3.40)	0.5634
**Marital Status** Single **vs.** Married	0.3900	0.4159	1.477	(0.65, 3.34)	0.3484
**Marital Status** Widowed **vs.** Married	−0.4699	0.9956	0.625	(0.09, 4.40)	0.6369
**Employee** Yes **vs.** No	−0.7084	0.3110	0.492	(0.27, 0.91)	0.0228
**Education Level** General education **vs.** University education	0.2055	0.2843	1.228	(0.70, 2.14)	0.4697
**Education Level** High education **vs.** University education	2.0668	0.8001	7.899	(1.65, 37.90)	0.0098
**Relationship W/Patient** Brother/Sister **vs.** Father/Mother	0.4146	0.4576	1.514	(0.62, 3.71)	0.3649
**Relationship W/Patient** Grandmother/Grandfather **vs.** Father/Mother	−0.4272	0.5679	0.652	(0.21, 1.99)	0.4519
**Relationship W/Patient** Husband/Wife **vs.** Father/Mother	2.1022	0.6493	8.184	(2.29, 29.22)	0.0012
**Relationship W/Patient** Other **vs.** Father/Mother	0.2945	0.6099	1.342	(0.41, 4.44)	0.6293

Probability modeled is caregiver burden = “Yes”.

## Data Availability

The data presented in this study can be obtained by contacting the corresponding author if desired.
